# Dyke-Davidoff-Masson Syndrome Presenting With Recurrent Seizures and Left-Sided Motor Weakness: A Case Report

**DOI:** 10.7759/cureus.107570

**Published:** 2026-04-23

**Authors:** Muthukumarasamy Thiyagarajan, Adithya Mani, Gowrisankar Kugalur Saravanan

**Affiliations:** 1 Medicine, Stanley Medical College, Chennai, IND; 2 Medicine, Coimbatore Medical College, Coimbatore, IND

**Keywords:** antiepileptic drugs, cerebral hemiatrophy, developmental delay, dyke-davidoff-masson syndrome, hemiparesis, neuroimaging, pediatric neurology, seizures

## Abstract

This study aimed to highlight that, regardless of a condition's rarity, every patient seeks timely recognition and effective management, underscoring clinicians' responsibility to remain vigilant and informed to provide optimal care. Dyke-Davidoff-Masson syndrome, first described in 1933, represents a classic radiological and clinical manifestation of this condition. This syndrome is characterized by unilateral cerebral atrophy accompanied by compensatory skull thickening and hyperpneumatization of the paranasal sinuses. The underlying cerebral insult may occur during fetal development or early childhood, and based on the timing of injury, the condition can be categorized as congenital or acquired. Various etiologies have been implicated, including vascular insults, trauma, infections, neoplasms, and idiopathic causes. Neuroimaging plays a crucial role not only in establishing the diagnosis but also in providing insight into the timing and extent of the cerebral injury.

We report a case of a 15-year-old male with a history of recurrent focal seizures since the third day of life, developmental delay, and left-sided motor weakness. He had been on long-term antiepileptic therapy and presented with a breakthrough seizure following brief non-adherence to medication. Radiological evaluation revealed classical features of right cerebral hemiatrophy, along with other findings confirming the diagnosis of Dyke-Davidoff-Masson syndrome. This case highlights the classical clinical presentation and imaging findings of a rare syndrome and emphasizes the importance of early recognition, strict adherence to antiepileptic drugs, caregiver counseling, and supportive rehabilitation to improve long-term functional outcomes in affected individuals.

## Introduction

Dyke-Davidoff-Masson syndrome (DDMS), also known as cerebral hemiatrophy, is a rare neurological condition characterized by underdevelopment of one cerebral hemisphere following a significant cerebral insult. It most commonly involves the left hemisphere and shows a male predominance, as observed in the present case [1,2]. The apparent hemispheric predilection has been hypothesized to relate to differences in cerebral perfusion during early brain development; however, the exact mechanism remains unclear [3]. DDMS can be congenital, resulting from in utero insults, or acquired due to post-natal injury. Reported etiologies include vascular insults, space-occupying lesions, infections, and idiopathic causes [1,2]. The severity of neurological deficits depends on the extent and timing of the insult. Clinical manifestations commonly include recurrent seizures, hemiparesis or hemiplegia, facial asymmetry, and delayed developmental milestones [1,4]. In some cases, however, the presentation may be subtle and detected incidentally on imaging.

Magnetic resonance imaging (MRI) is essential for diagnosis, as it demonstrates both the extent of cerebral injury and associated compensatory changes. Typical findings include unilateral cerebral hypoplasia, ex vacuo dilatation of the ipsilateral ventricles, hyperpneumatization of the frontal sinus, ipsilateral shift of the falx cerebri, skull thickening, and prominent cortical sulci [4,5].

Management is primarily symptomatic and supportive. Treatment includes seizure control with antiepileptic drugs (AEDs), physiotherapy, occupational therapy, and management of associated psychiatric symptoms when present. In patients with drug-resistant seizures, hemispherectomy may be considered as a potential therapeutic option [6]. Similar clinical and radiological findings have been described in previously reported cases of Dyke-Davidoff-Masson syndrome [7,8]. This case is noteworthy due to the rare presentation of Dyke-Davidoff-Masson syndrome and highlights the importance of considering it in the differential diagnosis of cerebral hemiatrophy, particularly when clinical findings are subtle.

## Case presentation

History of present illness

A 15-year-old male presented with a complaint of a single episode of seizure occurring the previous night. He had been on antiepileptic drugs (AEDs) since early childhood, but reported non-compliance for the past two days due to running out of medication. The seizure episode was characterized by brief jerky movements involving the left upper and lower limbs, along with the left side of the face. This was associated with deviation of the angle of the mouth to the left and repeated blinking movements of both eyes. There was no history of tongue bite. Notably, the episode included inappropriate laughter and concluded with hand-clapping movements. His current medications included oral sodium valproate 400 mg twice a day, clobazam 10 mg at bedtime, and levetiracetam 500 mg twice a day.

Medical history

The patient had a history of recurrent seizure episodes requiring multiple hospital admissions and evaluations. He was diagnosed with Dyke-Davidoff-Masson syndrome at five years of age based on radiological findings.

Birth and developmental history

The patient was born to parents in a second-degree consanguineous marriage and is the fourth child in the family. He was delivered via normal vaginal delivery and cried immediately after birth. He was breastfed, and the mother reported regular antenatal care, although no records were available. On the third day of life, he developed focal seizures involving the left upper and lower limbs, requiring admission to the neonatal intensive care unit (NICU) and initiation of AED therapy, which was continued for one month. Developmental milestones were delayed, and he was not attending school. However, he was able to perform activities of daily living independently.

Neurological examination

On examination, he was thin-built and poorly nourished. There was no facial asymmetry, and obvious wasting of the left upper and lower limbs was noted. His cranial nerves were intact except for the facial nerve. He was noted to have weakness of orbicularis oris and oculi over the left side.

Summary of motor, sensory, and cerebellar findings

Table 1 presents the muscle bulk of the upper and lower limb groups measured in centimeters, demonstrating reduced bulk on the left side consistent with disuse atrophy. There was hypotonia of the left upper and lower limbs, with normal tone on the right. Muscle power in each upper and lower limb muscle group was assessed using the Medical Research Council (MRC) grading system and is summarized in Table 2. Deep tendon and superficial reflexes are summarized in Table 3, with no significant differences between the two sides. All sensory modalities were found to be intact. Examination of cerebellar function was normal, indicating that the cerebellar hemispheres were well developed, and the patient demonstrated an independent, stable gait without swaying or risk of falling.

**Table 1:** Motor examination - bulk.

Muscle bulk	Left side	Right side
Upper limb (arm)	21 cm	23 cm
Upper limb (forearm)	18 cm	20 cm
Lower limb (thigh)	31 cm	33 cm
Lower limb (calf)	26 cm	27 cm

**Table 2:** Motor examination - power (Medical Research Council scale).

Region	Muscle group/movement	Left side	Right side
Upper limb	Shoulder (flexion, extension, abduction, adduction)	4/5	5/5
	Elbow (flexion, extension)	4/5	5/5
	Wrist (flexion, extension)	4/5	5/5
	Hand (intrinsic muscles)	4/5	5/5
Lower limb	Hip (flexion, extension, abduction, adduction)	4/5	5/5
	Knee (flexion, extension)	4/5	5/5
	Ankle (dorsiflexion, plantar flexion)	4/5	5/5
	Toes (flexion, extension)	4/5	5/5

**Table 3:** Motor examination - reflex.

Deep tendon reflex (DTR)	Left side	Right side
Biceps	2+	2+
Triceps	2+	2+
Brachioradialis	2+	2+
Patellar	2+	2+
Achilles	2+	2+
Superficial reflex	Left side	Right side
Corneal	Normal	Normal
Conjunctival	Normal	Normal
Abdominal	Symmetric	Symmetric
Cremasteric	Present	Present
Plantar reflex	Plantar flexion	Plantar flexion

Investigations

**Figure 1:**
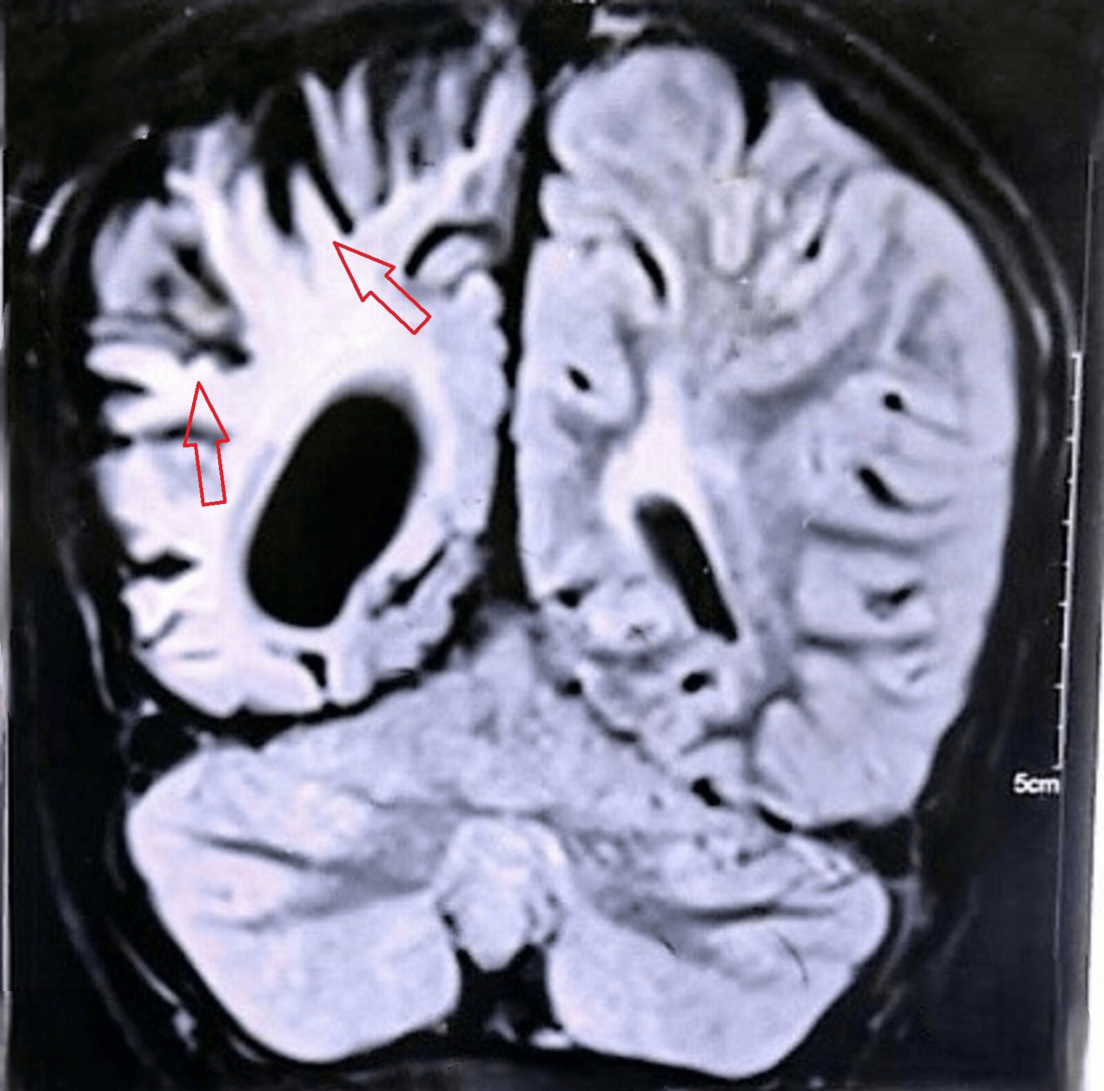
Hypoplasia of the right cerebral hemisphere with prominent sulci (arrows).

**Figure 2:**
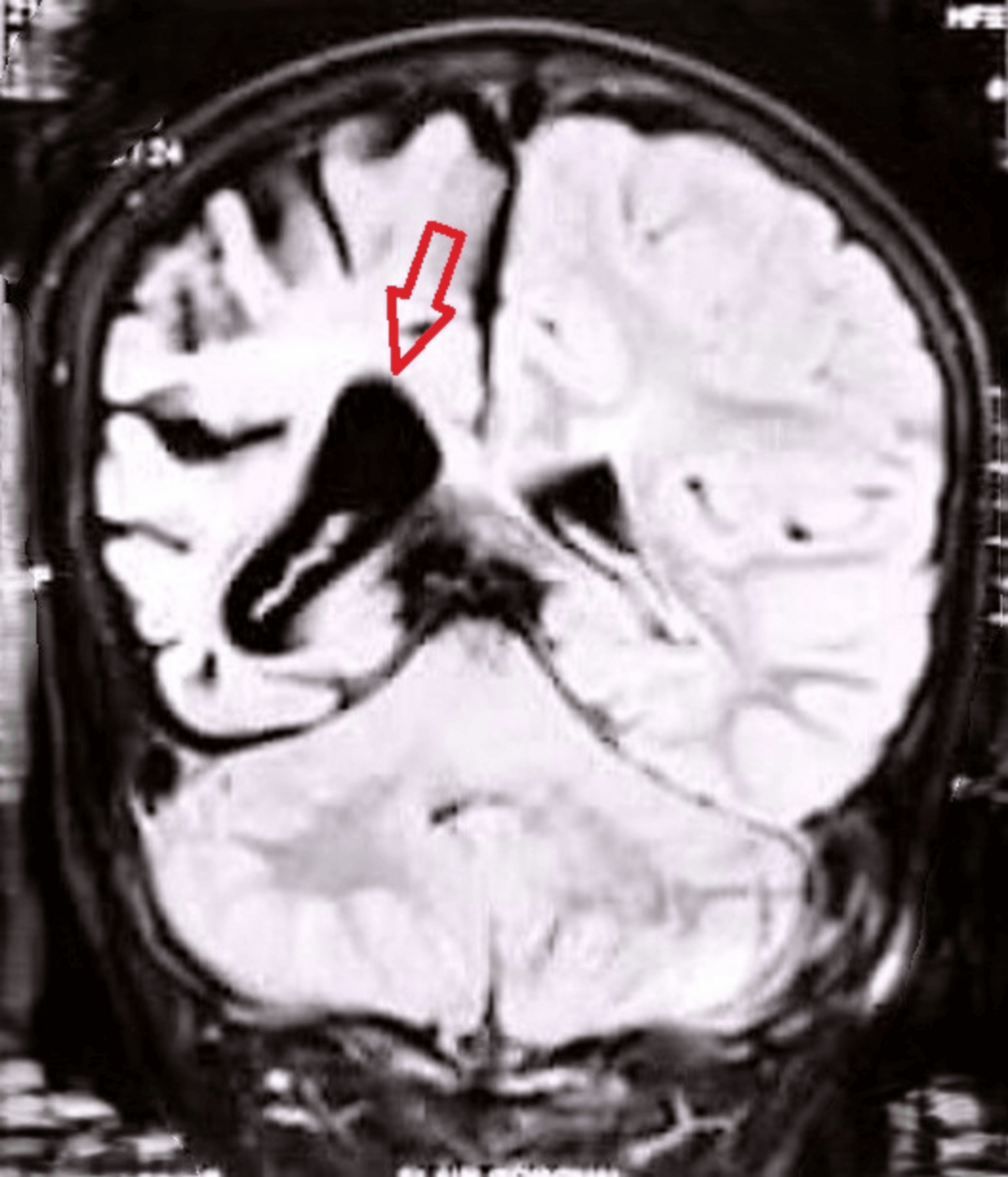
Relative enlargement of the right lateral ventricle compared to the left (arrow).

**Figure 3:**
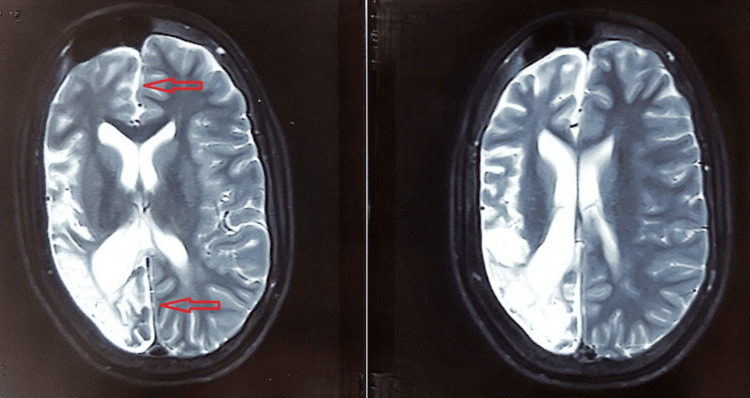
Mild rightward deviation of the falx cerebri (arrows).

**Figure 4:**
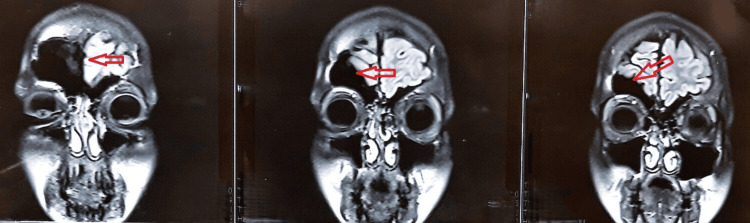
Marked hyperpneumatization of the right frontal sinus (arrows).

**Figure 5:**
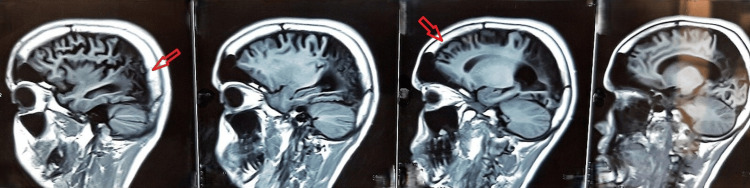
Thickening of the right-sided skull over the hypoplastic right cerebral hemisphere (arrows).

Electroencephalography (EEG) demonstrated focal epileptiform discharges arising from the affected hemisphere, along with background slowing, consistent with an underlying structural brain abnormality. Magnetic resonance imaging (MRI) was performed to evaluate the structural abnormalities of the brain. These imaging findings from Figures 1-3 were obtained at the time of initial diagnosis, approximately 10 years ago, and provide baseline information for longitudinal assessment of the patient’s neurological status. Together, they highlight the classical structural features associated with cerebral hemiatrophy in Dyke-Davidoff-Masson syndrome. Figure 1 shows hypoplasia of the right cerebral hemisphere, characterized by poorly developed gyri and prominent sulci, indicative of underdevelopment of the cortical architecture. Figure 2 demonstrates ex vacuo dilatation of the ipsilateral lateral ventricle, reflecting loss of cerebral parenchyma, while the cerebellar hemispheres appear unaffected, suggesting selective involvement of the cerebral hemisphere. Figure 3 illustrates a mild deviation of the falx cerebri toward the right side, consistent with the asymmetric cerebral growth. The following figures were obtained during the current hospital admission. Figure 4 shows hyperpneumatization of the right frontal sinus. Figure 5 demonstrates increased thickness of the right-sided skull.

Treatment

The patient was admitted and started on IV AED initially with IV levetiracetam 500 mg BID, and later put on oral AED as follows: carbamazepine 400 mg twice a day, levetiracetam 500 mg twice a day, sodium valproate 400 mg twice a day, clobazam 10 mg at bedtime, and folate 5 mg once a day. He was observed for five days during which he was seizure-free. Counseling regarding medication adherence and prognosis was explained to the caregiver (mother) before discharge.

Discharge and follow-up

The patient was discharged after four days of hospitalization and remained clinically stable. At two-week follow-up, he remained clinically stable with no recurrence of seizures.

## Discussion

This case provides important longitudinal follow-up data in a patient with previously diagnosed Dyke-Davidoff-Masson syndrome, highlighting both the clinical course and radiological stability over a 10-year period. The current admission, prompted by a breakthrough seizure due to medication non-compliance, allowed for reassessment and comparison with prior imaging, which demonstrated no significant interval changes. This supports the non-progressive nature of the condition [1].

Dyke-Davidoff-Masson syndrome (DDMS) is a rare condition resulting from early cerebral insult, leading to hemiatrophy and compensatory cranial changes [1,2]. The pathophysiology is closely related to the timing of the insult; when injury occurs during intrauterine life or early childhood, incomplete brain development results in reduced intracranial pressure and subsequent compensatory osseous changes, including skull thickening, hyperpneumatization of the frontal sinus, and ipsilateral midline shift [1,4]. In this patient, the presence of seizures as early as the third day of life strongly suggests a congenital or perinatal insult.

Clinically, DDMS commonly presents with recurrent seizures, hemiparesis, facial asymmetry, and cognitive impairment [1,6]. However, the degree of neurological deficit varies depending on the extent and timing of the insult [6]. In contrast to many reported cases with severe disability, our patient demonstrates relatively preserved functional independence despite long-standing disease.

Neuroimaging plays a central role not only in diagnosis but also in longitudinal assessment [1,4]. Previous CT-based studies have demonstrated features such as unilateral cerebral atrophy, ipsilateral skull thickening, sinus hyperpneumatization, ventricular dilatation, and midline shift sufficient for diagnosing Dyke-Davidoff-Masson syndrome [7]. In the present case, MRI was preferred for seizure evaluation, although CT remains useful acutely to exclude intracranial hemorrhage immediately after a seizure episode.

While most reports emphasize imaging findings at initial presentation, this case uniquely demonstrates long-term structural stability, with no progression of cerebral atrophy or compensatory changes over a decade. This reinforces that DDMS is a static encephalopathy rather than a progressive condition [1].

Seizures remain the most disabling component of DDMS and require long-term management. In this case, the breakthrough seizure was most likely attributable to poor medication adherence rather than disease progression, underscoring the importance of patient and caregiver education. In refractory cases, surgical options such as hemispherectomy have shown favorable outcomes in selected patients [6,8]. Although DDMS is commonly associated with recurrent seizures, the clinical course can be variable, and prolonged seizure-free periods have been reported in certain cases [9].

Differential diagnoses include Rasmussen encephalitis, Sturge-Weber syndrome, and post-traumatic cerebral atrophy. Rasmussen encephalitis typically demonstrates progressive neurological decline with refractory seizures and evolving hemispheric atrophy; however, our patient showed no evidence of progressive deterioration, and serial imaging over a 10-year period demonstrated stable findings, making this diagnosis unlikely [10].

Sturge-Weber syndrome is characterized by leptomeningeal angiomas, cortical calcifications, and often associated cutaneous vascular malformations such as facial port-wine stains. These features were absent in our patient, and no evidence of intracranial vascular malformations was identified on imaging [11]. Post-traumatic cerebral atrophy was also considered; however, there was no history of prior head trauma. The presence of compensatory skull thickening and sinus hyperpneumatization in this patient strongly supports the diagnosis of Dyke-Davidoff-Masson syndrome.

Prognosis depends on the timing and severity of the initial insult, with relatively better outcomes reported in patients presenting after two years of age [12]. Early initiation of supportive therapies, including physiotherapy and occupational therapy, is essential for optimizing functional independence [13].

## Conclusions

Dyke-Davidoff-Masson syndrome should be considered in patients presenting with long-standing seizures, hemiparesis, and developmental delay, particularly when characteristic radiological features are identified. This case highlights the importance of correlating clinical findings with neuroimaging and demonstrates the long-term structural stability of the condition despite ongoing seizure activity.

The present report underscores key clinical lessons, including the critical role of medication adherence in preventing breakthrough seizures and the value of early diagnosis and sustained multidisciplinary management. Timely recognition, appropriate antiepileptic therapy, caregiver education, and early rehabilitation are essential in optimizing functional outcomes and improving quality of life. Increased awareness of this rare condition among clinicians can facilitate prompt diagnosis and effective long-term care.
